# A Novel *Exophiala* Species Associated With Disseminated Granulomatous Inflammation in a Captive Eastern Hellbender (*Cryptobranchus alleganiensis alleganiensis*)

**DOI:** 10.3389/fvets.2020.00025

**Published:** 2020-01-31

**Authors:** Cynthia Hopf, Erin A. Graham, Connie F. C. Gibas, Carmita Sanders, James Mele, Hongxin Fan, Michael M. Garner, Nathan P. Wiederhold, Robert Ossiboff, Noha Abou-Madi

**Affiliations:** ^1^Department of Clinical Sciences, Cornell University College of Veterinary Medicine, Ithaca, NY, United States; ^2^Department of Comparative Diagnostic and Population Medicine, University of Florida College of Veterinary Medicine, Gainesville, FL, United States; ^3^Fungus Testing Laboratory & Molecular Diagnostics Laboratory, Department of Pathology and Laboratory Medicine, University of Texas Health Science Center at San Antonio, San Antonio, TX, United States; ^4^Northwest ZooPath, Monroe, WA, United States; ^5^Department of Population Medicine and Diagnostic Sciences, Cornell University College of Veterinary Medicine, Ithaca, NY, United States

**Keywords:** amphibian, chromoblastomycosis, chromomycosis, cryptobranchid, exophialosis, fungus, phaeohyphomycosis, salamander

## Abstract

The genus *Exophiala* is composed of ubiquitous, pigmented, saprotrophic fungi and includes both terrestrial and waterborne species. Though *Exophiala* species are generally considered opportunistic pathogens, exophialosis can be an important cause of morbidity and mortality in aquatic and semi-aquatic species. Over a 6-year period, a captive 32-year-old male eastern hellbender (*Cryptobranchus alleganiensis alleganiensis*), was treated for recurring, slow growing, ventral midline cutaneous masses. Excisional biopsies were characterized histologically by granulomatous dermatitis with low numbers of intralesional, pigmented fungal conidia and hyphae. Bacterial and fungal cultures of the masses and skin were negative on two separate submissions. Polymerase chain reaction amplification of a short fragment of the fungal 28S large subunit (LSU) ribosomal RNA was positive with 100% nucleotide sequence identity to several species of *Exophiala*. Following recurrence after successive rounds of antifungal therapy, euthanasia was elected. At necropsy, similar dermal granulomatous inflammation and intralesional pigmented fungal elements as observed in excisional biopsies formed a thick band in the dermis and extended through the coelomic body wall. Visceral dissemination was noted in the lung and kidney. Postmortem DNA sequence analysis of a large portion of the fungal LSU as well as the internal transcribed spacer (ITS) from a portion of frozen affected dermis identified the fungus as a novel species, *Exophiala* sp. 1 (UTHSCSA R-5437).

## Introduction

Native to the United States, hellbenders (*Cryptobranchus alleganiensis*) are large aquatic salamanders represented by two subspecies: the eastern hellbender (*C*. *a*. *alleganiensis*) and the Ozark hellbender (*C*. *a*. *bishopi*). *Cryptobranchus a. alleganiensis* ranges from New York to Georgia, through Tennessee and the Ohio River Valley to the Ozarks and inhabit clear, swift-flowing streams with rocky bottoms (1). Wild populations have been declining due to historical overharvesting for the pet trade, and are now threatened by habitat deterioration and infectious disease outbreaks (*Batrachochytrium dendrobatidis* [*Bd*]) (1–4). The eastern hellbender was designated as a species of special concern in New York State in 1983 and is listed as near-threatened by the IUCN Red List. Conservation efforts have focused on head-start and reintroduction programs, habitat restoration, and disease surveillance (1, 5). Some reported health problems in the eastern hellbender include traumatic injuries, neoplasia (epidermal papilloma, squamous cell carcinoma, Sertoli cell tumor, and poorly differentiated sarcoma), and infections with *Saprolegnia* or *Bd* (1).

Phaeohyphomycosis is a disease caused by a heterogenous group of septate dark-walled phaeoid fungi that can cause both subcutaneous and systemic infections. Over 100 species of melanized fungi are in this category, including *Exophiala* and *Cladophialophora*, that both belong to the order *Chaetothyriales* (6). *Exophiala* species can be opportunists or pathogens of immunocompetent humans and have also caused disease in fish, amphibians, and invertebrates (7). Study of the taxonomy and clinical disease characteristics of *Exophiala* species is ongoing with updates in their classification. This report characterizes a unique morphologic presentation of fatal phaeohyphomycosis in an eastern hellbender associated with an undescribed species of *Exophiala*.

## Case Presentation

A 32-year-old male eastern hellbender (*C*. *a*. *alleganiensis*), wild caught as a juvenile, was examined following the discovery of a chain of small, nodular masses noted along the ventral midline. The masses were slow-growing and initially involved only the skin. Two excisional surgeries were performed 31 months apart due to the recurrence of the condition.

For surgical procedures the hellbender was anesthetized in a bath solution of 0.1% MS222 (Ethyl 3-aminobenzoate methanesulfonate, Millipore Sigma, Darmstadt, Germany) buffered with sodium bicarbonate. Induction was achieved after 15 min and anesthesia was maintained by covering the body of the hellbender with gauzes soaked with a buffered 0.05% solution of MS222. Heart rate was monitored using a Doppler ultrasonic flow detector and depth of anesthesia was gauged by the lack of righting reflex and response to surgical stimulation. No increase in the concentration of MS222 was required throughout the procedure. For both anesthetic procedures, the hellbender recovered completely after 2 fresh water bath changes and 20 min after the anesthesia was discontinued.

During the first surgery, the ventral midline masses involved only the skin. The larger masses were excised for diagnostic purposes. Post-operative treatment included enrofloxacin (Baytril 22.7 mg/ml, Bayer, Shawnee Mission, Kansas 66201 USA) 5 mg/kg given intra-coelomically. Butorphanol (0.5 mg/L) bath solution (Dolorex 10 mg/ml, Merck, Madison, New Jersey, 07940 USA) was used for analgesia for the first 24 h. Eleven days post-operatively, the skin wound dehisced over 2–3 mm but the coelomic wall closure remained intact and the skin healed by second intention without further complication. Microscopically the lesions consisted of dermal granulomatous inflammation. A Fite's acid fast stain revealed no acid fast positive bacteria within the lesions. A Gomori methenamine silver (GMS) stain identified scattered and infrequent spherical yeast-like structures that were difficult to find in foci of inflammation. Culture and PCR were not attempted at this time and treatment was declined as euthanasia was considered. The hellbender healed and no recrudescence of the masses was noted for several years.

Three and a half years after the initial surgery, new masses were discovered, and a second surgery was performed. The masses had invaded into the coelomic wall. They were excised using radiosurgery and blunt dissection in the anesthetized animal. The chain of masses removed measured 9 × 4.5 × 2 cm. Sections of the mass were submitted for aerobic bacterial culture, fungal culture, and histologic analysis.

**Figure 1:**
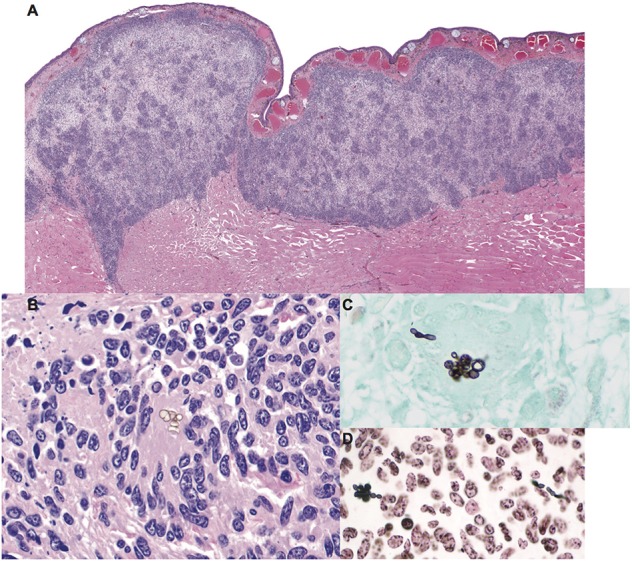
Granulomatous mycotic dermatitis in an eastern hellbender (*Cryptobranchus alleganiensis alleganiensis*). **(A)** The dermis is expanded by a band of nodular to diffuse granulomatous inflammation with focal epidermal attenuation and extension through underlying striated muscle fibers. Hematoxylin and eosin (HE). **(B)** Small, sporadic, clusters of extracellular pigmented conidia are surrounded by numerous epithelioid macrophages and multinucleated giant cells amid scattered pyknotic and karyorrhectic cellular debris (HE). **(C)** Fungal elements are argyrophilic; conidia are accompanied by rare hyphae with thin, non-parallel walls and sporadic septation. Gomori methenamine silver (GMS). **(D)** Conidia and hyphae exhibit positive cytochemical reactivity within the fungal wall, consistent with a pigmented (melanin) fungus. Fontana-Masson.

Cultures for bacteria and fungi were negative. Histologic findings were similar to the previous biopsy and were characterized by granulomatous dermatitis with epidermal ulceration, edema, and hyperplasia (Figures 1A,B). Low numbers of pigmented, round to oval-shaped conidia (6.5 to 8.5 μm diameter) were present in small clusters within granulomatous foci (Figure 1B). Rare non-branching hyphae with thin, non-parallel, pigmented walls ranging from 5 to 10 μm in diameter and sporadic septation were also noted. A GMS stain revealed that the conidia and hyphae were intensely argyrophilic (Figure 1C) and demonstrated positive cytochemical reactivity with a Fontana-Masson stain for melanin, consistent with a pigmented fungus (phaeohyphomycosis) (Figure 1D). No acid-fast bacteria were highlighted with Ziehl-Neelsen or Fite-Faraco staining.

Nucleic acids were extracted from scrolls of paraffin-embedded skin using a commercially available kit (DNeasy Blood and Tissue Kit, QIAGEN, Germantown, Maryland, USA), and fungal DNA was amplified by polymerase chain reaction (PCR) using universal primers that target a variable region within the large 28S subunit (8). An amplicon of approximately 260 bp was sequenced bidirectionally (Genomics Facility, Biotechnology Resource Center, Cornell University), assembled and edited of primer sequence (Geneious R10; Auckland, New Zealand) for a final product size of 224 bp, and compared for homology with sequences in GenBank (https://www.ncbi.nlm.nih.gov/genbank/). The resultant product exhibited 100% nucleotide identity to a number of *Exophiala* species, including *Exophiala opportunistica* (KP347962.1), *E*. *bonariae* (KR781083.1), and *E*. *cancerae* (KF928502.1).

Post-operative treatment consisted of 5 mg/kg of enrofloxacin injected subcutaneously (SC) in a pocket of electrolyte solution (Normosol-R, Hospira Inc, Lake Forest, IL 60045 USA), and meloxicam (Metacam 5 mg/ml, Boehringer Ingelheim, St. Joseph, MO 64506 USA) 0.2 mg/kg also given SC every other day for 8 and 6 days respectively. In light of the histology and PCR results, itraconazole (Sporanox, 10 mg/ml, Janssen Pharmaceutica N.V. Beerse, Belgium) was added at a dose of 5 mg/kg once daily hidden in the prey food item. A total of only 18 treatments of itraconazole over the course of 30 days were successfully administered due to hypophagia and poor compliance. Itraconazole was discontinued after the 18th successful administration. The surgical wound healed without complication. The hellbender's appetite improved. Two years after the second surgery, the masses recurred at the same location with ulceration of the skin. Due to the recurrence of the lesions and the extent of the masses, euthanasia was elected.

## Post-Mortem Diagnostics

**Figure 2:**
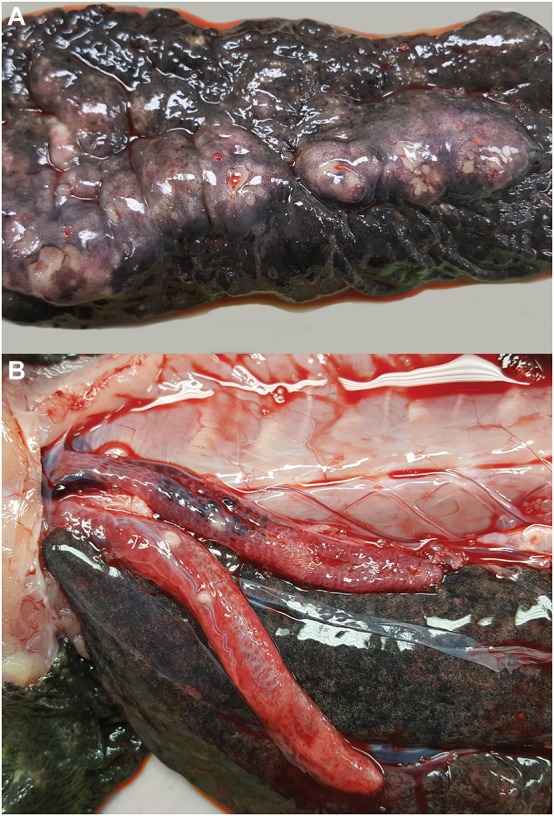
Multicentric granulomatous mycotic inflammation in an eastern hellbender (*Cryptobranchus alleganiensis alleganiensis*). **(A)** The ventral dermis is expanded by multinodular coalescing granulomas with ulceration of the overlying epidermis. **(B)** Similar nodular granulomas are present within the lungs.

Gross necropsy revealed a chain of multinodular, coalescing, and ulcerated dermal masses of the ventral coelomic body wall (Figure 2A). The masses had a gross appearance similar to those of previous surgical excisions, and were concentrated in the region of the two previous surgical incisions. The masses extended through the body wall and were multifocally visible through the coelomic mesothelium. Multiple, white-tan nodular masses were also present in the lungs (Figure 2B) and the kidneys. No gross abnormalities were noted in other visceral organs. Several tissues were sampled and fixed in 10% neutral buffered formalin for microscopic examination and frozen for further testing, including the body wall sections of granulomatous lesion, sections of skin and mass, left distal thoracic limb, eye, brain, heart, urinary bladder, lungs, liver, spleen, kidneys, pancreas, intestines, segments of small and large intestine, and testicular tissue.

Histologic examination confirmed the presence of generalized granulomatous dermatitis associated with pigmented fungal elements as noted in historical excisional biopsies (Figures 1A–D). Granulomatous inflammation extended into the muscle of the coelomic body wall, but did not penetrate into the coelomic cavity. Evidence of visceral mycotic dissemination was noted in the lung and kidney and was characterized by moderate to severe granulomatous pneumonia and nephritis with low numbers of intralesional pigmented fungi as described in the skin lesions.

A 1 cm^3^ sample of frozen, affected dermis collected at the time of necropsy was lysed in a Precellys^®^ Evolution tissue homogenizer (Bertin Technologies, Montigny-le-Bretonneux FRANCE) and genomic DNA was extracted using EZ1 DNA tissue kit with a BioRobot EZ1 instrument (QIAGEN) following manufacturer's instructions. PCR amplification of the internal spacer region (ITS) and partial large subunit gene of the nuclear rDNA (LSU) were done using primers specifically designed for *Exophiala*: EXO1 and EXO2 (9), and U1 and U2 (8), respectively. Sequencing was done with the same primers using BigDye Terminator v. 3.1 Cycle Sequencing Kit (Applied Biosystems, Foster City, CA, USA). The sequences generated were deposited in GenBank, https://www.ncbi.nlm.nih.gov/ with the accession nos. MK253014 (ITS) and MK253015 (LSU).

Using the obtained ITS sequence, identification was done by an in-house BLASTn search within a sequence dataset compiled in Bioedit v7.0.5 program comprising short barcode identifiers located in the ITS2 region of reference and authentic strains of *Exophiala* and related species (10, 11). A 100% match was not found with any of the strains in the dataset. Subsequently, BLASTn searches in GenBank were performed using partial ITS and partial LSU sequences. The top ITS results showed that the isolate (UTHSCSA R-5437) matched 100% with an unidentified *Exophiala* sp. S4.3 (KY322615), 96% with *E*. *cancerae* CBS 120420^T^ (NR_1377664), 95.69% with *E*. *opportunistica* CBS 109811^T^ (KF928435) and 95.32% with *E*. *bonariae* CBS 139957^T^ (NR_144964). In the partial LSU BLASTn search, a 100% match was observed with *E*. *cancerae* (KF928502), and type strains of *E*. *opportunistica* (KF928501) and *E*. *bonariae* (KR781083), 98-99% matches with *E*. *psychrophila* CBS 191.87^T^ (MH873750), *E*. *salmonis* CBS 157.67^T^ (MH870616), *E*. *radicis* P2854^T^ (KT723448) and *E*. *pisciphila* CBS 537.73^T^ (MH870790). Phylogenetic analysis was then conducted using only the ITS sequence because the LSU sequence obtained was short and was unreliable, and repeat sequencing of LSU failed to obtain a longer sequence for use.

The ITS sequences of representative *Exophiala* species and relatives were obtained from GenBank based on BLASTn searches and on the datasets of Yong et al. (12) and Borman et al. (13). The sequences were assembled and aligned using MUSCLE (14) as implemented in Sequencher version 5.4.6-Build 46289 (Gene Codes Corp., MI USA) and refined visually using SE-AL (15). The best fit evolutionary model for this dataset was determined using the Find Best DNA Models for maximum likelihood (ML) as implemented in MEGA version 7 (16). General Time Reversible model with Gamma distributed rate variation and an estimated proportion of invariable sites (GTR+G+I) (17) was found to be the best fit evolutionary model for this dataset having the lowest Bayesian Information Criterion score (BIC) (18). Maximum likelihood analysis was conducted with GTR+G+I substitution model and 1000 bootstrap (19) iterations. Bayesian inference was done using MrBayes v3.2.5 (20) applying the same substitution model. The Markov Chain Monte Carlo (21) started from a random tree topology and lasted 6 million generations, where every 100th tree was retained and the first 25% of the trees were discarded as burn in. A 50% majority rule consensus tree and posterior probabilities were calculated. Bayesian posterior probabilities (BPP) > 0.90 and bootstrap (BT) values > 70% were considered significant.

**Figure 3:**
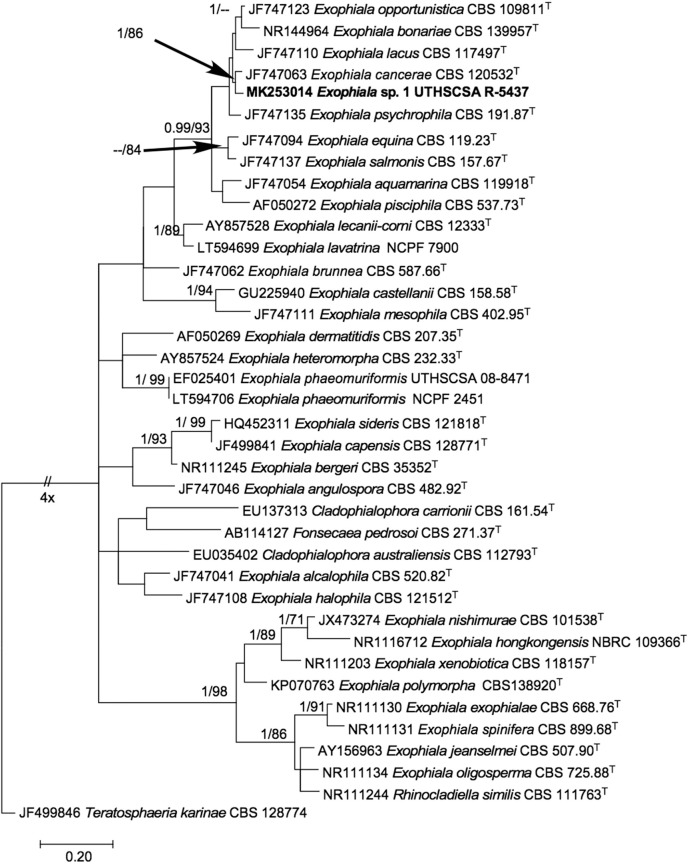
Phylogenetic tree inferred from ITS sequences showing the relationship of *Exophiala* sp. UTHSCSA R-5437 to known *Exophiala* species. Branch lengths are proportional to phylogenetic distance. ML bootstrap support values (1000 resampling, Right) >70% and posterior probability values from Bayesian inference (Left) > 0.90 are shown above the branches. *Teratosphaeria karinae* CBS 128774^T^ was designated as outgroup. ^T^, type strain; CBS, CBS-KNAW Fungal Biodiversity Center culture collection, The Netherlands; NBRC, National Biological Resource Center, Japan; NCPF, UK National Collection of Pathogenic Fungi, PHE UK National Mycology Reference Laboratory and School of Biological Sciences, University of Bristol, Bristol, United Kingdom; UTHSCSA, Fungus Testing Lab, University of Texas Health Science Center at San Antonio, Texas USA. Sequence accession numbers are indicated in front of species names.

The phylogenetic tree inferred from maximum likelihood (Figure 3) showed our isolate within a monophyletic clade (BPP 0.99/ BT 93%) comprising mostly of waterborne species, i.e., *E*. *opportunistica, E*. *lacus, E*. *cancerae, E*. *psychrophila, E*. *salmonis, E*. *aquamarina, E*. *pisciphila* (7) and other species originally isolated from non-aquatic sources, i.e., *E*. *bonariae* (22) and *E*. *equina* (7). Within the clade, it is closest to *E*. *cancerae* with high support (BPP 1.00 / BT 86%). ITS pairwise comparison of UTHSCSA R-5437 with *E*. *cancerae* CBS 120420^T^ (NR_1377664) however, showed 15 nucleotide base pair differences.

## Discussion

Phaeohyphomycosis is caused by pigmented filamentous fungi including *Exophiala* species (23). Waterborne *Exophiala* species are important pathogens in fish including *E. salmonis* in cut-throat trout (*Salmo clarkii*) and the Atlantic salmon (*Salmo salar*). *Exophiala pisciphila* caused an epizootic in the channel catfish (*Ictalurus punctatus*), and brain and skin lesions in smooth dogfish (*Mustelus canis*) (7). In amphibians, infection with *Exophiala* are reported in marine toads (*Bufo marinus*) and European blind cave salamanders (*Proteus anguinus*) (24). Case reports of dermal or disseminated disease in reptiles are described in a Galapagos tortoise (*Geochelone nigra*) (25), Eastern box turtle (*Terrapene carolina carolina*) (26), and Aldabra tortoise (*Geochelone gigantean*) (27). *Exophiala dermatitidis* and *E*. *oligosperma* have both been reported to cause infections in humans (7).

Melanized fungi including *Exophiala* spp. can cause disease more frequently in aquatic animals than in terrestrial animals. Further, dry, thicker skin, the presence of fur or feathers, and the absence of sweat glands are thought to provide a degree of protection against infection in reptiles, birds, and mammals (7). Infections may occur secondary to traumatic percutaneous inoculation (23, 27, 28). Immunosuppression may be a factor in producing infection, but phaeohyphomycosis has been reported in immunocompetent animals (7, 27–29).

Pathogenicity associated with *Exophiala* is not described in *C*. *a*. *alleganiesis*. The source of infection in this case is unknown but may be percutaneous invasion of the fungus associated with ventral abrasions. Concurrent diseases that may have contributed were not identified in this animal. It is considered possible that the fungus of this report was a primary pathogen.

The initially observed nodular dermatitis spread to deeper structures of the body wall and eventually spread systemically. Over time lesions were identified in the dermis, skeletal muscle of the body wall, lungs, and kidneys. The lesions in the viscera suggested hematogenous spread from the skin. The lesions in this animal were first noted 6 years prior to the first surgery and had not appeared to change, until a progressive growth was noted. No observed changes in the hellbender's environment or health justified the change in the masses. Detailed reports of lesions from *Exophiala* infections are rare and include systemic mycosis with *E. angulospora* in an Atlantic halibut (*Hippoglossus hippoglossus*), and disseminated mycosis in a colony of Ornate-horned frogs (*Ceratophrys ornata*) with cutaneous and visceral lesions (28, 29); however, *Exophiala* species infections are commonly encountered in fish and amphibians during routine diagnostic investigations of captive collections (personal observations, MG).

Diagnostic techniques used in cases of phaeohyphomycoses include impression smear cytologic exam, histologic exam, culture, molecular identification, and phylogenetic analysis (23, 28, 29). In the present case, the fungus was identified histologically followed by molecular screening. Initial antemortem PCR results on a short segment of the 28S ribosomal large subunit identified the species as an *Exophiala*. Differentiation from other closely related species was not possible based on the short size and conserved nature of the targeted region. Attempts to culture the organism were unsuccessful. Different *Exophiala* species have similar morphologic and physiologic characteristics and can be difficult to differentiate based on clinical signs, gross necropsy, and histologic examination. The organism of this report had unique features in histologic section that differed considerably from the more commonly observed infections. In hematoxylin and eosin stained sections of infected tissue, *Exophiala* typically forms long, streaming, yellow, slender hyphae and occasional branching elements with parallel cell walls. In this salamander, the lesions were chronic, with minimal necrosis, and the fungal agent was present in low numbers primarily as small yeast-like or septate conidial forms, with only rare hyphae observed. In this regard, infection with this organism in hellbenders or other species could possibly misconstrued as a different fungus, or be overlooked due to the low density of organism in tissue sections.

Identification of *Exophiala* species is challenging because they are highly pleomorphic and produce synanamorphs in their life cycles (10, 30, 31). Although these species are generally recognizable as *Exophiala*, identification is difficult using morphological and other phenotypic criteria because almost identical structures are observed even with phylogenetically distantly related species (10). DNA sequencing and the increasing availability of sequences in public databases (e.g., GenBank) improved fungal identification and led to the discovery of novel species as well as reclassification of species in their natural groupings. ITS sequences in *Exophiala* and related species have been found to have variabilities caused by stutter formation in PCR products making this target unreliable for identification (10). However, short fragments in the ITS2 region have differences in nucleotide composition that are stable allowing them to be used as barcodes by clinical diagnostic laboratories for the identification of medically important black yeasts and relatives (10). In this case, the barcode did not give a species identity because the *Exophiala* strain in this study did not match with any of the species in the database and could represent a new species. ITS phylogenetic analysis showed that this sequence is closely related with *E*. *cancerae* (BPP1.00/ BT 86%); however pairwise ITS sequence comparison with that of *E. cancerae* showed 15 base pair differences. Based on the in-house Bioedit barcode identifier sequence BLASTn result, BLASTn results in GenBank and phylogenetic analysis, this organism is as a putatively novel *Exophiala* species closely related to *E*. *cancerae*. Confirmation and description of this organism as a new species is not possible at this time because of the lack of viable culture of the organism and failure to sequence other gene loci for a multi-gene phylogenetic analysis.

Treatment options for *Exophiala* species are limited. In humans, this includes radiation therapy, amputation, resection followed by skin grafts, and systemic administration of amphotericin B and 5-fluorocytosine (32). Ketoconazole and acridine dyes combined with excisional surgery were unsuccessful in a marine toad (*Bufo marinus*) (33). Treatment was successful in an Aldabra tortoise (*Geochelone gigantean)* infected with *Exophiala oligosperma* (27). In the present case, two surgeries with aggressive debridement of the masses, followed by oral itraconazole failed to resolve the infection. Without a culture, the appropriateness of itraconazole therapy could not be confirmed. Poor compliance may have been caused by post-operative stress, the increased feeding frequency to match the treatment schedule, or a side effect of the drug. Decreased frequency of the treatment was attempted, but the hellbender remained hypophagic and the treatment was ultimately discontinued. Treatment in bath water might have been successful and was considered but declined due to husbandry considerations.

## Data Availability Statement

The datasets generated for this study can be found in the GenBank, https://www.ncbi.nlm.nih.gov/.

## Ethics Statement

The care and treatment of this animal, as well as the testing performed post-mortem were approved by the Rosamond Gifford Zoo (Syracuse, NY, USA) Animal Care Committee and the Rosamond Gifford Zoo (Syracuse, NY, USA) IACUC committee.

## Author Contributions

CH, and NA-M performed the medical and surgical management of this case, the submission of the samples to necropsy, writing of the manuscript. CG, CS, JM, HF, and NW performed the molecular and genetic analysis on the samples provided. MG, RO, and EG processed the tissues for histopathologic examination, and performed the analysis of the results. All authors discussed the results and commented on the manuscript.

### Conflict of Interest

The authors declare that the research was conducted in the absence of any commercial or financial relationships that could be construed as a potential conflict of interest. The handling Editor declared a past co-authorship with one of the authors RO.
